# Targeting lysine acetyltransferase 6A/B in cancer: a conceptual and translational review of epigenetic regulation, immune microenvironment remodeling, and the dual-compartment hypothesis

**DOI:** 10.3389/fimmu.2026.1961903

**Published:** 2026-10-06

**Authors:** Cong Ma, Jingru Wang, Guanhua Qiao, Xinhong Wu, Hongmei Zheng

**Affiliations:** Breast Cancer Center, Hubei Cancer Hospital, Tongji Medical College, Huazhong University of Science and Technology, Wuhan, Hubei, China

**Keywords:** conceptual framework, dual-compartment hypothesis, epigenetic regulation, KAT6A/B targeting, translational medicine, tumor immune microenvironment

## Abstract

While lysine acetyltransferase 6A (KAT6A; MOZ/MYST3) and 6B (KAT6B; MORF/MYST4) function as context-dependent oncogenic drivers—with KAT6B also displaying tissue-specific tumor-suppressive roles—their pharmacological targeting holds promise as a therapeutic avenue. This conceptual and translational review synthesizes evidence from genetic depletion models, pharmacological catalytic inhibitors, and emerging proteolysis-targeting chimera (PROTAC) degraders, introducing the “Dual-Compartment Hypothesis” as a conceptual interpretive framework to organize current evidence and generate testable hypotheses regarding their putative impacts on the tumor immune microenvironment (TIME). Under this framework, KAT6A/B blockade within the primary tumor compartment is proposed to indirectly foster regional antitumor immunity via preclinically implicated pathways, including tumor cell ferroptosis, cyclic GMP-AMP synthase–stimulator of interferon genes (cGAS-STING) activation, and interleukin-6 (IL-6) suppression, alongside putative downstream influences on immune checkpoints. In contrast, systemic drug exposure in the secondary compartment may directly alter hematopoiesis and lymphocyte development, creating a tension between local immune activation and systemic constraints. Importantly, immune cells within the tumor microenvironment may also be directly suppressed by KAT6A/B inhibition—an alternative outcome that remains to be systematically investigated. Resolving these divergent outcomes necessitates distinguishing genetic knockout/knockdown (KO/KD) from pharmacological inhibition or degradation—a distinction that is critical because much of the mechanistic evidence derives from genetic models, while accounting for KAT6A versus KAT6B isoform selectivity. Finally, we outline testable translational frameworks, such as intermittent dosing schedules and rational immunotherapeutic combinations, to direct subsequent experimental and clinical validation, while acknowledging that current early-phase clinical observations (objective response rate [ORR]/clinical benefit rate [CBR]) reflect composite therapeutic responses and require further paired biomarker validation to definitively isolate immune-mediated effects.

## 1 Introduction

Non-mutational epigenetic reprogramming represents a fundamental hallmark of cancer, wherein its biochemical reversibility provides a strong theoretical basis for targeted chromatin interventions and differentiation therapies (1). Histone acetylation relaxes chromatin structure by neutralizing the positive charges on lysine residues, subsequently recruiting bromodomain-containing effectors to regulate chromatin accessibility and transcriptional programs (2).

While first-generation epigenetic agents—primarily histone deacetylase (HDAC) inhibitors—demonstrated clinical utility, their application in solid tumors remains constrained by off-target toxicity and narrow therapeutic windows. To overcome these limitations, drug discovery efforts have shifted toward histone acetyltransferases (HATs), which directly catalyze acetyl group transfer and frequently display lineage-specific amplification or genomic alteration across human cancers (3).

Pan-cancer genomic profiling highlights KAT6A and KAT6B as recurrently altered in diverse histologies (4). While KAT6A and KAT6B predominantly function as context-dependent oncogenic drivers, their biological roles are remarkably nuanced—with KAT6B also exhibiting tissue-specific tumor-suppressive functions depending on the cellular lineage and epigenetic landscape. In hematological malignancies, KAT6A acts as a key driver of acute myeloid leukemia (AML), where oncogenic fusion proteins—such as KAT6A-CREB-binding protein (KAT6A-CBP) and KAT6A-transcriptional intermediary factor 2 (KAT6A-TIF2)—aberrantly recruit co-activators to sustain stemness genes including homeobox A9 (HOXA9) and Meis homeobox 1 (Meis1) (5–7). In solid tumors, KAT6A maps to the 8p11-p12 amplicon in luminal B breast cancer, where focal copy-number gain sustains clonogenic capacity and predicts late disease recurrence (8). KAT6A amplification similarly correlates with adverse clinical outcomes in uterine serous carcinoma (9), whereas the KAT6A-TFE3 fusion defines a distinct driver event in MiT family translocation renal cell carcinoma (10).

KAT6B mirrors these oncogenic functions in specific contexts while displaying distinct lineage selectivity. In AML, the KAT6B-NUT midline carcinoma family member 2B (KAT6B-NUTM2B) fusion drives congenital myeloid sarcoma via hyperactivation of the HOXA gene cluster (11). In solid tumors, KAT6B maintains estrogen receptor (ER) expression to facilitate breast cancer progression, with elevated expression conferring poor prognosis in early-stage disease (12). Furthermore, in tongue squamous cell carcinoma, KAT6B promotes chemoresistance and upregulates immunosuppressive factors through the phosphoinositide 3-kinase (PI3K)/protein kinase B (Akt)/nuclear factor kappa B (NF-κB) signaling axis (13). On the other hand, loss-of-function events and epigenetic silencing of KAT6B have been documented to facilitate tumorigenesis in distinct tissue contexts, emphasizing the necessity of evaluating KAT6A/B targeting within specific genomic and lineage backgrounds. These context-dependent roles, together with the broader physiological functions of KAT6 family members across tissues, have been systematically reviewed (14).

To exploit these vulnerabilities, small-molecule catalytic inhibitors and PROTAC degraders targeting KAT6A/B have recently entered clinical evaluation (15, 16). In addition to tumor-intrinsic cell-cycle arrest and senescence, emerging evidence indicates that targeting KAT6A/B alters the TIME (17, 18). However, translating these findings hinges on a clean line between genetic KO/KD and pharmacological catalytic inhibition or degradation. Genetic models remove the protein entirely—destroying its non-catalytic scaffolding alongside enzymatic activity—whereas small-molecule catalytic inhibitors preserve these structural hubs (19). Furthermore, while trade-offs between local and systemic effects are common in oncology, KAT6A/B stands out: a single agent concurrently sparks intratumoral immune activation and provokes systemic hematopoietic suppression. We emphasize that this spatial framework is proposed to organize current evidence and generate testable hypotheses, not to represent a verified biological dichotomy; direct suppression of intratumoral immune cells remains a formally possible alternative. In this conceptual and translational review, we systematically evaluate current KAT6A/B-targeting strategies, detail their immunomodulatory impacts, and propose the “Dual-Compartment Hypothesis” as a conceptual interpretive framework to guide testable translational regimens and rational combination immunotherapies.

## 2 Medicinal chemistry advancements in KAT6 inhibitors and targeted degraders

Targeting KAT6A and KAT6B relies on exploiting their conserved domain architecture and multimeric assembly mechanisms. The catalytic MYST domain mediates acetyl transfer through a ping-pong mechanism: acetyl-coenzyme A (Ac-CoA) binds within a conserved pocket, enabling an active-site cysteine to perform a nucleophilic attack that forms a covalent acetyl-enzyme intermediate, which subsequently transfers the acetyl group to target histone lysine residues (20). This catalytic cycle provides distinct structural sites for pharmacological intervention: the Ac-CoA binding pocket for competitive small-molecule inhibitors, the catalytic cysteine residue for targeted covalent inhibition, and subtle structural variations in the binding pocket that dictate isoform selectivity—not only between KAT6A and related MYST family members like KAT7, but also between KAT6A and KAT6B homotypic paralogs despite their high sequence homology.

In addition to enzymatic catalysis, KAT6A and KAT6B act as epigenetic readers through tandem plant homeodomain (PHD) fingers (PHD1–PHD2). Structural analyses indicate that PHD2 anchors the unmodified histone H3 tail via a conserved PxGxW motif, an interaction sterically inhibited by H3K4 trimethylation. Upon H3K9 or H3K14 acetylation, the carbonyl oxygen of the acetyl group inserts into a hydrophobic pocket in PHD1, increasing binding affinity and positioning the target lysine toward the catalytic core (21, 22). This coupled reader-writer feedback loop stabilizes complex localization at transcriptionally active chromatin loci. Accordingly, small molecules designed to allosterically disrupt this recognition interface represent a non-catalytic strategy to displace KAT6A/B from chromatin without necessarily abolishing catalytic acetyltransferase activity.

Optimal enzymatic activity requires KAT6A/B monomers to assemble into a tetrameric complex with the scaffold protein bromodomain and PHD finger-containing protein 1 (BRPF1) and the subunit effectors inhibitor of growth family member 5 (ING5) and ESA1-associated factor homolog 6 (EAF6). BRPF1 serves as a critical structural scaffold, interacting via KAT6A/B residues to stabilize the catalytic conformation—enhancing enzymatic activity over sevenfold—and target the complex to promoter regions (23, 24). Given this obligate assembly, targeting the protein–protein interaction (PPI) interfaces or inhibiting the BRPF1 bromodomain (e.g., with GSK6853) provides a means to disrupt the KAT6A/B transcriptional complex (25). These structural interaction surfaces offer molecular entry points for targeted degradation technologies, including PROTACs and chemical inducers of proximity (CIPs). Unlike catalytic small-molecule inhibitors that leave the physical complex intact, PROTAC degraders deplete the entire protein, removing non-catalytic scaffold roles, liquid–liquid phase separation capacity, and chromatin-tethering functions alongside enzymatic activity (19). Systematic chemoproteomic profiling has further revealed that commonly used KAT6A/B tool compounds, including WM-1119 and WM-3835, exhibit hierarchical selectivity across MYST family members and may display unexpected cross-reactivity (26, 27). These findings reinforce the importance of distinguishing pharmacological inhibition from genetic depletion in mechanistic studies.

### 2.1 Discovery and optimization of acylsulfonylhydrazide-based inhibitors

High-throughput screening targeting the MYST catalytic domain initially identified CTX-0124143 as an Ac-CoA-competitive hit, though low potency and unfavorable pharmacokinetic properties limited its utility (28). Structural optimization produced WM-8014, an acylsulfonylhydrazide-based pan-KAT6A/B catalytic inhibitor. Functioning as a cell-permeable pyrophosphate mimic, WM-8014 occupies the Ac-CoA binding pocket to competitively inhibit enzymatic acetyl transfer. As a reversible catalytic inhibitor, WM-8014 abolishes enzymatic acetyltransferase activity while leaving the physical assembly of the tetrameric KAT6–BRPF1 complex and its non-catalytic chromatin-tethering functions intact. However, high lipophilicity, extensive plasma protein binding, and rapid metabolic clearance restricted WM-8014 primarily to *in vitro* mechanistic evaluations (29).

To overcome these pharmacokinetic limitations, aza-heterocyclic substitution of the core scaffold yielded WM-1119. This structural modification lowered lipophilicity and extended metabolic half-life while retaining nanomolar binding affinity, oral bioavailability, and dual pan-KAT6A/B selectivity. In *Eμ-Myc* lymphoma models, WM-1119 established *in vivo* proof-of-concept for pharmacological KAT6A/B inhibition, demonstrating that enzymatic catalytic blockade is sufficient to induce cellular senescence and arrest tumor growth (30, 31). Because WM-1119 does not discriminate between KAT6A and KAT6B, dual-paralog inhibition presents distinct translational liabilities. Systemic pan-KAT6A/B blockade exacerbates suppression of normal myeloid hematopoiesis in the secondary compartment, whereas unselective KAT6B inhibition risks blunting its lineage-specific tumor-suppressive functions or inducing compensatory microenvironmental adaptations—effects that are further contextualized within the dual-compartment framework discussed in Section 3.5.

To mitigate these pan-inhibitory liabilities, subsequent optimizations focused on achieving paralog selectivity. Linker modification led to derivative 6J, which extends into the binding pocket to form a hydrogen bond with the Ser690 side chain of KAT6A, conferring selectivity for KAT6A over KAT6B and KAT8 (28). Although derivative 6J exhibits antiproliferative activity and oral bioavailability in preclinical models, its full *in vivo* immunomodulatory profile and capacity to spare hematological toxicity relative to pan-KAT6A/B inhibitors remain to be defined in testable translational settings—a distinction that underscores the importance of isoform-selective targeting strategies discussed in **Table 1**.

**Table 1:** Dual-compartment immunomodulatory mechanisms and translational caveats of KAT6A/B interventions.

Mechanism	Evidence level	Intervention type	Key references	Experimental system	Primary immunological outcome & phenotype	Translational caveats
I. Tumor-intrinsic immunogenicity and antigenicity						
Ferroptosis & DAMP release	B	shRNA KD (KAT6A)	(17, 45)	Colon/pancreatic cancer lines; syngeneic mouse models (*in vitro* + *in vivo*); Measured: lipid ROS, ICD markers	Downregulates GPX4/SLC7A11, induces lipid ROS accumulation and ICD	Requires pharmacological validation with small-molecule inhibitors; DCs/NK cells are highly susceptible to lipid peroxidation
cGAS-STING axis activation	B/A	shRNA KD (KAT6A) + WM-1119 (pan-KAT6A/B)	(18)	Colorectal cancer lines; xenograft models (*in vitro* + *in vivo*); Measured: cytosolic dsDNA, IFN-I, CD8^+^ T cell infiltration	Induces DNA damage, triggers cGAS-STING-dependent IFN-I response and CD8^+^ T cell infiltration	KAT6A/KAT6B may exert opposing effects; isoform-selective tools required
Surface antigen exposure (GD2)	A	KAT6A/B catalytic inhibitor	(46) [Preprint]	Neuroblastoma cell models (*in vitro*); Measured: surface GD2 density, ADCC/CAR-T killing	Derepresses ST8SIA1/B4GALNT1, increases surface GD2 density, enhances ADCC/CAR-T clearance	Preprint requires peer review; kinetics of antigen loss upon drug withdrawal need *in vivo* definition
II. Reversal of evasion and microenvironmental remodeling						
PD-L1 transcriptional suppression	A	WM-3835 (KAT6A) + CRISPR/dCas9	(47, 48)	Colorectal/endometrial cancer lines; murine syngeneic models (*in vitro* + *in vivo*); Measured: H3K23pr ChIP, CD274 mRNA, GZMB/perforin	Erases H3K23pr, silences CD274 transcription, restores T cell GZMB/perforin secretion	Compensatory TIM-3/LAG-3 upregulation observed in select models; biomarker monitoring recommended
Paracrine c-KIT network blockade	B	Kat6a KO	(49)	Syngeneic solid tumor models (*in vivo*); Measured: KIT transcription, TGF-β/IL-10, DC maturation	Represses KIT transcription, reduces TGF-β/IL-10 secretion, unblocks DC maturation	c-KIT expressed in HSCs; systemic suppression may limit therapeutic window
TAM repolarization (SMAD3 axis)	B/A	shRNA KD (KAT6A) + WM-1119 (pan-KAT6A/B)	(50)	Macrophage co-cultures; TNBC mouse models (*in vitro* + *in vivo*); Measured: SMAD3 acetylation, CD206/Arg1/MHC-II/iNOS, IL-6/IL-22/TNF-α, MDSC influx	Blocks SMAD3 acetylation, suppresses IL-6/IL-22/TNF-α, repolarizes suppressive TAMs (CD206^+^/Arg1^+^) toward a pro-inflammatory/phagocytic phenotype (MHC-II^+^/iNOS^+^), reduces MDSC influx by 60–70%	Discordance between acute repolarization and long-term macrophage homeostasis remains unresolved
Biphasic SASP regulation	A	WM-1119 (pan-KAT6A/B)	(30)	Eμ-Myc lymphoma model (*in vivo*); Measured: SASP factors, pro-inflammatory TAM/NK dynamics, MDSC/Treg dynamics	Early phase: pro-inflammatory TAMs and NK cells for tumor clearance; Late phase: MDSC/Treg recruitment and T cell exhaustion	Demands precise intermittent dosing schedules; risk of co-inhibiting potential KAT6B tumor-suppressive/immune-maintaining functions with pan-inhibitors
III. Secondary resistance, direct intratumoral suppression and systemic toxicity (negative feedback)						
Compensatory ERBB2 activation	A	PF-9363 (KAT6A/B)	(51, 52)	ER^+^/HER2^+^ breast cancer models (*in vitro*); Measured: ESR1-PAX2 binding, ERBB2 expression, MHC-I	ESR1 loss releases PAX2, driving ERBB2 activation, MHC-I downregulation, MDSC recruitment	HER2^-^ tumors lack this mechanism; baseline stratification required
Bystander T cell lipid toxicity	C	Indirect inference (ferroptosis-linked ROS)	(53, 54)	CD8^+^ T cell–tumor co-cultures (*in vitro*); Measured: CD36, lipid ROS, FAO	Tumor lipid ROS upregulates CD36 in TILs, impairs FAO, accelerates terminal exhaustion	Lacks direct KAT6A/B interventional data; requires combination with CD36/ACC inhibitors
Myeloid hematopoiesis suppression	A	PF-07248144 (pan-KAT6A/B)/PRT13722 (KAT6A degrader)	(15, 41) [Abstract]	Phase I clinical trials; CFU-GM progenitor assays (Human + *in vitro*); Measured: Neutrophil counts (Grade ≥3), CFU-GM colonies	Pan-inhibition causes Grade ≥3 neutropenia in 37.2% of patients, whereas selective degradation spares CFU-GM progenitors	Risk of co-inhibiting potential KAT6B tumor-suppressive functions with pan-inhibitors; establishes clinical rationale for degrader strategies or intermittent schedules
Lymphocyte development & metabolic arrest	B/A	Kat6a KO + WM-1119 (pan-KAT6A/B)	(55, 56)	Lymphocyte progenitors; purified T cells (*in vitro* + *in vivo*); Measured: Meis1/Hoxa binding, Hk2/Pkm promoter acetylation	Disrupts Meis1/Hoxa axis in B progenitors; represses Hk2/Pkm promoter acetylation in T cells	Continuous high-dose exposure impairs lymphocyte development; intermittent dosing or CD8-targeted degraders recommended

Evidence levels were assigned using an adapted GRADE framework, in which Grades A, B, and C denote moderate, low, and very low certainty of evidence, respectively. Risk of bias was incorporated as a domain of the adapted GRADE assessment.

### 2.2 Rational design and clinical translation of highly selective benzisoxazole-based inhibitors

Unlike acylsulfonylhydrazide derivatives developed through empirical structure-activity relationship (SAR) campaigns, the benzisoxazole class emerged from structure-based rational drug design. Optimization of the initial hit CTx-533 produced PF-9363, a highly potent pan-KAT6A/B catalytic inhibitor incorporating benzenesulfonamide and isoxazole scaffolds that mimic the diphosphate binding orientation of Ac-CoA. PF-9363 forms a key hydrogen bond with Ile649 in the KAT6A catalytic pocket, achieving sub-nanomolar potency and greater than 50, 000-fold selectivity over other MYST family members, such as KAT7 (32, 33). As a competitive catalytic inhibitor, PF-9363 selectively suppresses enzymatic acetyltransferase activity without disrupting the physical assembly of the multimeric KAT6 complex or its non-catalytic chromatin-tethering functions.

Based on this scaffold, PF-07248144 entered clinical evaluation as an oral pan-KAT6A/B catalytic inhibitor (34). In a first-in-human Phase I trial involving heavily pretreated ER^+^/HER2^-^ metastatic breast cancer, PF-07248144 demonstrated robust target engagement, inducing a sustained reduction in peripheral blood histone H3 lysine 23 acetylation (H3K23ac) levels (median inhibition of at least 70%) across all dose cohorts (35, 36). Dose-limiting toxicities (DLTs) were dominated by grade 3 or higher neutropenia (37.2%) (15), directly confirming the on-target requirement of KAT6A in normal hematopoietic progenitor maintenance. PF-07248144 has since advanced into a Phase III trial (KATSIS-1; NCT07062965) in HR^+^/HER2^-^ advanced breast cancer following prior CDK4/6 inhibitor-based therapy (37). This systemic myelosuppression in the secondary compartment underscores a fundamental safety bottleneck when considering combination strategies with immune checkpoint blockade (ICB).

Next-generation KAT6-targeting agents have evolved along two distinct clinical strategies: overcoming acquired resistance and refining isoform selectivity. To address resistance mechanisms, ISM5043 maintains high catalytic potency while demonstrating antitumor activity in patient-derived xenograft (PDX) models resistant to both endocrine therapy and CDK4/6 inhibitors (38); these data have been reported in conference presentations and company disclosures. In parallel, selective KAT6A inhibition is being actively explored to uncouple antitumor activity from the severe hematologic toxicity linked to dual pan-KAT6A/B blockade. Demonstrating this concept, the isoform-selective KAT6A inhibitor IST-477 exhibits preclinical monotherapy efficacy across ER^+^/HER2^-^ breast, ovarian, and esophageal cancer models (39); these findings are derived from preclinical company disclosures and require independent validation. Additional candidates—including OP-3136, KC1086, IDE574, and HLX97 (which initiated Phase 1 dosing in China in May 2026)—have entered early-phase clinical testing (**Table 2**), with available data currently limited to company disclosures, conference abstracts, and clinical trial registry entries, rather than peer-reviewed publications.

**Table 2:** Key pharmacological parameters and clinical progress of KAT6A/B inhibitors.

Category	Candidate	Target profile	Scaffold class	Potency (IC_50_/K_i_)	Key structural features & mechanism	Primary indication	Development stage
Tool Probes	CTX-0124143	KAT6A	Acylsulfonylhydrazide	~1.0 μM	Reversible Ac‐CoA competitive inhibition	—	Tool compound
	WM-8014	Pan‐KAT6A/B	Acylsulfonylhydrazide	8 nM (KAT6A)/28 nM (KAT6B)	Cell‐permeable pyrophosphate mimic; induces senescence	—	Tool compound
	CD-5	KAT6A (degrader)	PROTAC	85% max degradation	Selective cellular KAT6A degradation	—	Tool compound
Preclinical Candidates	WM-1119	Pan‐KAT6A/B	Acylsulfonylhydrazide	0.25 μM	1, 100‐fold vs. KAT5; 250‐fold vs. KAT7	Eμ‐Myc lymphoma	Preclinical
	6J	KAT6A (selective)	N‐(2‐oxoethyl) sulfonamide	30 nM	H‐bond with Ser690; spares KAT6B/KAT8	K562 leukemia	Preclinical
	PF-9363	KAT6A/B	Benzisoxazole	0.41 nM (KAT6A)/1.2 nM (KAT6B)	>50, 000‐fold vs. KAT7; anchors via Ile649	ER^+^/HER2^-^ breast cancer	Preclinical
	IST-477	KAT6A (isoform‐selective)	—	Not disclosed	Additive with CDK4/6 inhibition	ER^+^/HER2^-^ breast, ovarian, esophageal	Preclinical
Clinical‐Stage Candidates	PF‐07248144	KAT6A/B	Benzisoxazole	Sub‐nanomolar	≥70% H3K23ac reduction; DLT: neutropenia	ER^+^/HER2^-^ metastatic breast cancer	Phase III
	ISM5043	KAT6A/B	—	8 nM (KAT6A)/16 nM (KAT6B)	Synergizes with SOC in endocrine/CDK4/6i‐resistant models	Endocrine‐ and CDK4/6i‐resistant tumors	Phase I
	OP-3136	KAT6A/B	—	Nanomolar	OVCAR‐3 xenograft TGI = 104.5–105.4%	Advanced solid tumors	Phase I
	KC1086	KAT6/7 dual‐target	—	Nanomolar	Monotherapy TGI >90%; TGI = 101% with palbociclib	ER^+^/HER2^-^ breast cancer	Phase I
	IDE574	KAT6/7 dual‐target	—	Balanced inhibition	Equipotent KAT6/7 dual inhibitor; ~350‐ to 2, 000‐fold selective vs. KAT5/KAT8; targets 8p11 lineage addiction; durable PDX regression	8p11‐amplified breast, NSCLC, prostate, colorectal cancer	Phase 1
	HLX97	KAT6A/B	—	Not disclosed	Oral small-molecule KAT6A/B inhibitor	Advanced solid tumors	Phase I
Targeted Degraders	PRT13722	KAT6A (selective degrader)	PROTAC	Nanomolar degradation	Selective KAT6A degradation; spares CFU‐GM (mitigated hematotoxicity)	Advanced solid tumors	IND filing planned (mid-2026); Phase 1 expected (Q4-2026)

Blank cells (—) denote undisclosed scaffold structures or non-applicable primary indications. IDE574 (formerly IDE251): IND submitted December 2025; Phase I initiated April 2026 (NCT07540572). “Preclinical” denotes compounds with reported *in vivo* efficacy but not yet in human trials; Information for clinical-stage candidates and the targeted degrader is derived from clinical trial registries, conference abstracts, or company disclosures, as referenced in the main text; these data have not yet undergone full peer-reviewed publication.

### 2.3 Prospective exploration of novel drug modalities

While small-molecule catalytic inhibitors selectively block enzyme activity, occupying the Ac-CoA active site alone fails to disrupt non-catalytic scaffold roles—such as liquid–liquid phase separation (LLPS), chromatin tethering, and multimeric protein–protein interactions—nor does it eliminate the protein entity or prevent paralog-mediated functional compensation (19, 33, 40). Because genetic KO/KD eliminates the entire protein molecule, phenotypic discrepancies between genetic depletion and catalytic inhibition frequently emerge. These molecular constraints have catalyzed the development of novel drug modalities, particularly targeted protein degradation (TPD) and proximity-guided functional epigenetic rewriting.

Targeted protein degradation via PROTACs provides a mechanism to eliminate both catalytic and scaffolding roles of KAT6, effectively recapitulating the full biological impact of genetic loss-of-function phenotypes. The serendipitous identification of CD-5—a compound originally designed as a cyclin-dependent kinase 9 (CDK9) inhibitor—first demonstrated that KAT6A is vulnerable to ubiquitin–proteasome-mediated destruction (40). Subsequent rational engineering produced PRT13722, a highly potent and isoform-selective KAT6A degrader. By driving complete proteasomal clearance of the KAT6A protein entity rather than merely competing with Ac-CoA, PRT13722 achieves exquisite selectivity for KAT6A over KAT6B. In preclinical models, isoform-selective KAT6A degradation avoids the severe neutropenia associated with dual pan-KAT6A/B catalytic inhibition, establishing a key translational strategy to uncouple tumor-site efficacy from systemic hematological toxicity in the secondary compartment. An IND filing for PRT13722 is planned for mid-2026, with a Phase 1 trial expected to be initiated in the fourth quarter of 2026, pending regulatory clearance (41, 42)(company disclosure).

By contrast, emerging proximity-based modalities—such as acetylation-targeting chimeras (AceTACs) and transcriptional chemical inducers of proximity (TCIPs)—seek to repurpose intrinsic KAT6A/B acetyltransferase activity rather than eliminate the protein entity (43, 44). By utilizing non-catalytic domain binders while preserving enzymatic capability, these bifunctional molecules recruit KAT6A/B to specific genomic loci. Preclinical proof-of-concept studies suggest two primary translational applications: site-directed hyperacetylation of inactivated tumor suppressors to restore their expression, and targeted recruitment of KAT6A/B to promoters of epigenetically silenced genes to drive chromatin opening. Because catalytic turnover is preserved, sub-stoichiometric concentrations of redirected KAT6A/B can elicit robust downstream transcriptional activation.

These novel modalities also offer conceptual avenues to bypass the systemic immunosuppression inherent to global pan-KAT6A/B inhibition. Coupling degraders or CIPs with cell-type-specific delivery vehicles—such as antibody–degrader conjugates (ADCs/DACs) targeting lineage markers (e.g., CD8)—or restricting degradation to specific cell lineages could theoretically confine epigenetic remodeling to selected immune or tumor populations. This approach represents a proposed strategy to preserve systemic hematopoiesis while restoring regional antitumor immunity in the TIME. However, these proximity-based and cell-targeted platforms remain in early preclinical validation, requiring rigorous evaluation of their *in vivo* pharmacokinetics, tissue distribution, off-target vulnerabilities, and clinical feasibility.

## 3 Remodeling of the TIME and the “dual-compartment hypothesis” framework

The medicinal chemistry of KAT6A/B targeting has evolved from early acylsulfonylhydrazide probes to sub-nanomolar catalytic inhibitors and targeted degraders. Beyond suppressing tumor cell proliferation, these agents induce tumor-intrinsic stress that triggers damage-associated molecular pattern (DAMP) release, activates cGAS–STING signaling, and rewires immune checkpoint and myeloid cell networks to indirectly reshape the TIME.

Yet, the net immunological outcome of KAT6A/B inhibition reflects a “dual-compartment hypothesis” rather than simple immune activation or suppression. In the localized tumor microenvironment (the “first compartment”), tumor-intrinsic genomic instability and metabolic stress dominate. DAMPs released during ferroptosis, cytosolic DNA-induced cGAS–STING signaling, and altered immune checkpoint transcription promote local immunosurveillance through paracrine signaling and immunogenic cell death (ICD). In contrast, within systemic immunity (the “second compartment”), small molecules directly inhibit KAT6A/B in immune cells, altering Cd8 locus acetylation, disrupting CD4^+^ T-cell metabolic differentiation, and suppressing pro-inflammatory macrophage responses—a theme that is further explored in Section 3.5.

This tension between localized pro-inflammatory signaling and systemic immunosuppression is a central theme of the dual-compartment hypothesis and represents the principal translational barrier for immunotherapy combinations.

Sections 3.1–3.3 cover experimentally validated pathways of local immune activation (**Figure 1**, solid lines). Section 3.4 presents emerging hypotheses that lack direct pharmacological evidence (**Figure 1**, dashed lines). Finally, Section 3.5 integrates these local and systemic responses into a hypothesis-generating framework to examine how systemic costs dictate precision scheduling strategies.

**Figure 1:**
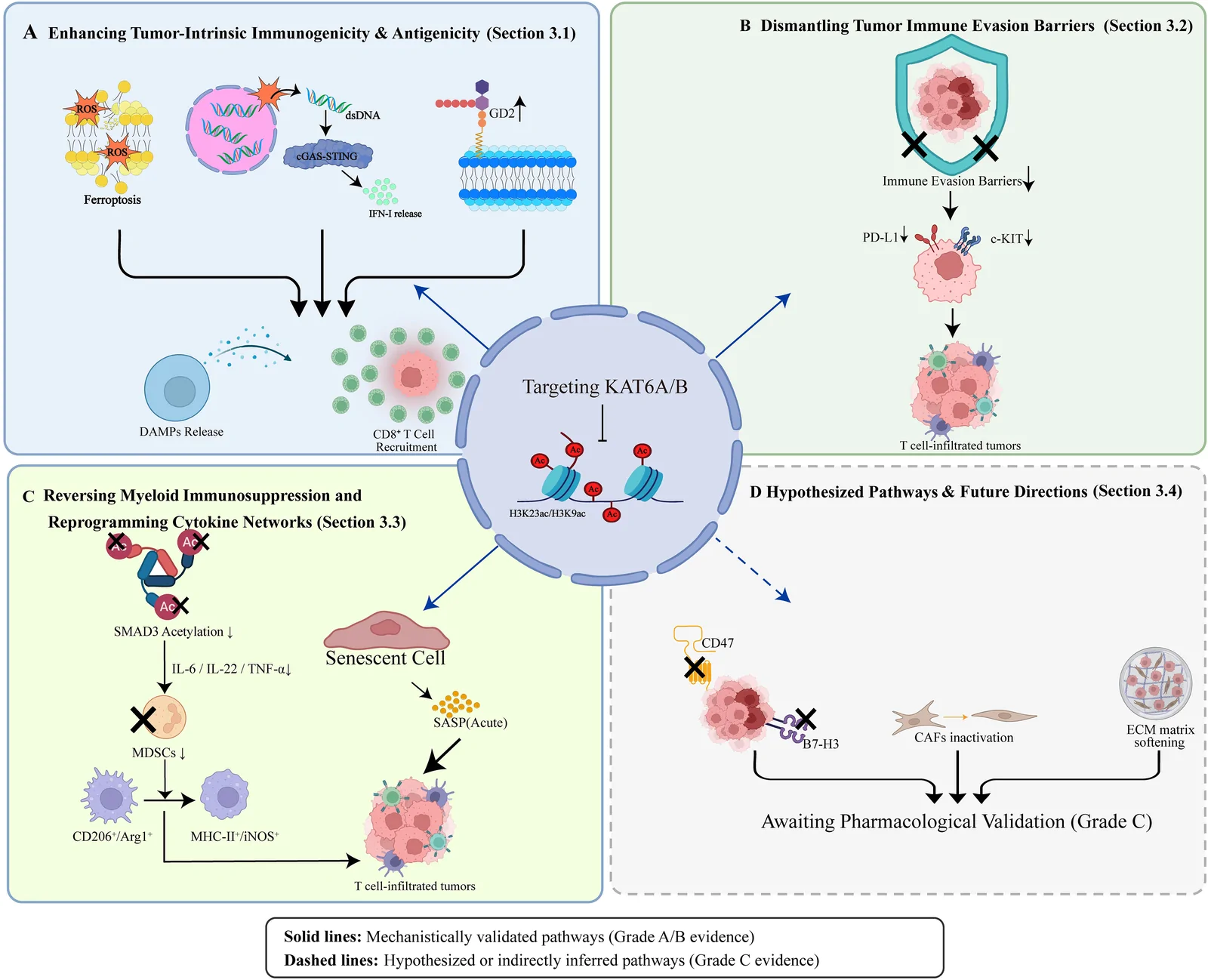
Schematic overview of KAT6A/B inhibitor-induced TIME remodeling. Targeting KAT6A/B depletes H3K23ac/H3K9ac, orchestrating immunomodulatory cascades across four main dimensions. **(A)** Enhancing tumor-intrinsic immunogenicity (Section 3.1): KAT6A/B targeting induces ferroptosis, activates the cGAS–STING pathway, and upregulates surface GD2 expression, promoting DAMP release and CD8^+^ T-cell recruitment. **(B)** Dismantling immune evasion barriers (Section 3.2): Small-molecule inhibition downregulates surface PD-L1 and c-KIT on tumor cells, converting non-inflamed tumors into T-cell-inflamed phenotypes. **(C)** Reversing myeloid immunosuppression (Section 3.3): Attenuation of SMAD3 acetylation reduces IL-6, IL-22, and TNF-α to deplete MDSCs. Concurrently, KAT6A/B targeting triggers acute cellular senescence and SASP secretion, driving macrophage repolarization from CD206^+^/Arg1^+^ to MHC-II^+^/iNOS^+^ phenotypes. **(D)** Hypothesized pathways and future directions (Section 3.4): Putative pathways awaiting direct pharmacological validation, including CD47/B7-H3 blockade, CAF inactivation, and ECM softening. (Solid lines: mechanistically validated pathways; Dashed lines: hypothesized pathways). Created with BioRender.com.

Importantly, much of the current mechanistic data relies on genetic KO/KD models rather than small-molecule inhibitors. Genetic depletion removes the entire KAT6A/B protein—including catalytic activity, scaffolding domains, and LLPS capacity—whereas catalytic inhibitors block Ac-CoA binding while preserving non-catalytic scaffolding roles. These functional differences can produce divergent biological phenotypes (Sections 5.1 and 5.3). To address this limitation, we apply an evidence grading system (**Table 1**) that distinguishes direct pharmacological validation from genetic inferential data across all subsections. Because the underlying data span diverse experimental models—from *in vitro* cell lines to *in vivo* animal systems and early-phase clinical trials—standard risk-of-bias frameworks (e.g., SYRCLE, GRADE) were not directly applicable in their original formats. Instead, we developed a simplified A–C grading system tailored to the KAT6A/B field and aligned it with an adapted GRADE framework: Grade A corresponds to moderate certainty (direct pharmacological validation in relevant models), Grade B to low certainty (genetic depletion models with indirectness), and Grade C to very low certainty (inference from upstream pathways without direct KAT6A/B intervention data).

Evidence Level Definitions:

A. Directly validated by pharmacological tools (small-molecule inhibitors/degraders)
B. Primarily supported by genetic KO/KD models
C. Indirect inference from other pathways, lacking direct KAT6A/B interventional data

Slash notation (e.g., “B/A”): Indicates that the same mechanism is supported by both genetic evidence (before slash) and pharmacological evidence (after slash). The overall evidence level is determined by the higher of the two (e.g., “B/A” is considered Level A overall). This notation is used to explicitly indicate that the conclusion is cross-validated by two independent lines of evidence, rather than relying on a single intervention modality.

References marked as [Preprint] or [Abstract] are cited to reflect rapid progress in this field; their conclusions have not yet undergone full peer review and are provided for reference only.

### 3.1 Enhancing tumor-intrinsic immunogenicity and antigenicity

In the TIME, KAT6A/B overexpression epigenetically silences tumor antigenicity and danger signaling, enforcing an immune-evasive phenotype. Targeting KAT6A/B restores tumor-intrinsic immunogenicity by inducing ICD, re-activating innate danger pathways, and upregulating surface antigens—priming malignant cells for effector T-cell recognition and elimination.

#### 3.1.1 Ferroptosis

Ferroptosis is a lipid peroxidation-driven, immunogenic cell death pathway with complex, context-dependent immunomodulatory effects. On one hand, ferroptotic tumor cells release DAMPs such as high-mobility group box 1 (HMGB1), which can promote dendritic cell (DC) maturation and antigen cross-presentation to initiate adaptive antitumor immunity (57, 58). On the other hand, microenvironmental ferroptotic stress and toxic lipid peroxides act as a double-edged sword: while transient oxidative stress may reprogram tumor-associated macrophages (TAMs) toward a pro-inflammatory phenotype, persistent accumulation of oxidized lipids directly impairs DC functional integrity, blunts natural killer (NK) cell cytotoxicity, and promotes exhaustion in tumor-infiltrating lymphocytes (TILs) (59). Consequently, the immunological trajectory of ferroptosis is dictated by its spatiotemporal dynamics and specific cellular context.

In colon cancer, KAT6A maintains H3K9ac at the GPX4 promoter to sustain its transcription; KAT6A KD downregulates GPX4, leading to lipid reactive oxygen species (ROS) accumulation, GSH depletion, Fe^2+^overload, and ferroptosis (17). In pancreatic cancer, circ_0005397 recruits KAT6A to the PCBP2 promoter to deposit H3K9ac and activate PCBP2 expression. PCBP2 then upregulates SLC7A11 and GPX4 while repressing ACSL4, suppressing ferroptosis. KAT6A KD or silencing circ_0005397 abolishes this protective axis and re-sensitizes cells to ferroptosis inducers (45). Because ACSL4 incorporates polyunsaturated fatty acids into membrane phospholipids to generate peroxidizable substrates, ACSL4 deficiency confers ferroptosis resistance (60).

KAT6B similarly protects against lipid peroxidation. In glioma, KAT6B binds the STAT3 promoter and deposits H3K23ac, promoting STAT3 transcription (61). STAT3 in turn upregulates antioxidant genes, including SLC7A11, GPX4, and FTH1; KAT6B KD disrupts this network and restores ferroptosis sensitivity (61, 62). STAT3 also influences ferroptosis through NCOA4-mediated ferritinophagy and ACSL4 transcription in a context-dependent manner (63, 64).

These metabolic responses likely feed into a reciprocal “immunity–ferroptosis” circuit. Activated CD8^+^ T cells secrete interferon-gamma (IFN-γ), which signals through JAK–STAT1 to repress SLC7A11 and SLC3A2 transcription. This impairs cystine import, depletes GSH, and compromises GPX4 activity—positioning IFN-γ as a metabolic sensitizer to ferroptosis. In clinical cohorts, low SLC7A11/SLC3A2 expression correlates with increased CD8^+^ T-cell infiltration, enhanced ferroptosis, and improved immunotherapy outcomes (53).

Notably, these findings rely heavily on genetic silencing models. Whether catalytic inhibitors (e.g., WM-1119, PF-9363) fully replicate these effects—or differ because scaffolding functions remain intact—requires validation using canonical ferroptosis readouts (Fe²^+^, GSH, MDA, and lipid ROS). Likewise, proving the operational role of the immunity–ferroptosis loop under KAT6A/B inhibition necessitates CD8^+^ T-cell depletion and IFN-γ neutralization *in vivo*. Finally, because GPX4 and intact antioxidant defenses are essential for CD8^+^ T-cell memory, NK cell survival, and DC function, unselective systemic GPX4 suppression may compromise central memory (T_CM_) and tissue-resident memory (T_RM_) T cells alongside tumor clearance. This stark contrast highlights the translational necessity of timing KAT6A/B inhibition to selectively induce tumor-intrinsic ferroptosis while sparing infiltrating effector leukocytes.

#### 3.1.2 Activating the cGAS–STING pathway and endogenous danger sensing

Targeting KAT6A/B activates cGAS–STING signaling through dual mechanisms: epigenetic desilencing and genomic stress, converting immunologically “cold” tumors into T cell-rich microenvironments.

Mechanistically, KAT6A overexpression maintains high H3K23ac levels at the DNMT1 promoter, recruiting c-Myc to drive DNMT1 transcription. Elevated DNMT1 hypermethylates the cGAS promoter, silencing innate DNA sensing. KAT6A KD downregulates DNMT1, triggering cGAS promoter demethylation and transcriptional restoration. This reactivates type I interferon (IFN-I) production and induces CXCL10 and CCL5 expression, establishing a chemotactic gradient for CD8^+^ T-cell recruitment. Consequently, genetic or pharmacological KAT6A modulation promotes CD8^+^ T-cell infiltration and synergizes with anti-PD-1 therapy in mouse tumor models (18).

In addition to transcriptional desilencing, KAT6A depletion triggers cytosolic DNA sensing by inducing genomic instability. In double-strand break repair, KAT6A recruits 53BP1 via H3K23ac deposition (65) and, independently of its catalytic activity, undergoes LLPS to form condensates that sequester repair factors such as PARP1 (19). Catalytic inhibitors, while blocking H3K23ac-dependent recruitment, cannot dismantle these LLPS-driven condensates—a limitation that may be overcome by protein degraders or genetic depletion, which eliminate the KAT6A protein entity entirely. Unrepaired DNA damage and chromosome missegregation lead to micronuclei formation (66). Micronuclear rupture releases double-stranded DNA into the cytosol, activating cGAS to synthesize cGAMP and trigger STING–TBK1 signaling (67, 68) —linking structural DNA damage directly to innate immune sensing.

STING activation subsequently restores tumor antigen presentation. Induced IFN-I binds IFNAR in a paracrine fashion, upregulating cell-surface major histocompatibility complex class I (MHC-I) molecules—a key step for T-cell recognition and lysis (68). STING also regulates MHC-I expression through interferon-independent mechanisms, such as ISG15 signaling (69). Re-establishing the STING–MHC-I axis enhances tumor-infiltrating lymphocyte (TIL) antigen recognition and amplifies perforin- and granzyme-mediated cytotoxicity.

#### 3.1.3 Exposing surface antigens

Disialoganglioside (GD2) is a neuroectoderm-derived tumor glycolipid with restricted normal tissue expression. In neuroblastoma, KAT6A/B inhibition reduces global H3K23ac, yet derepresses the rate-limiting GD2 synthetic enzymes ST8SIA1 (GD3 synthase) and B4GALNT1 (GD2 synthase), likely through the loss of core transcriptional repressors. The resulting surge in cell-surface GD2 increases target antigen density, which enhances natural killer (NK) cell-mediated antibody-dependent cellular cytotoxicity (ADCC) and improves the cytotoxicity of GD2-targeted CAR T-cell therapies at low effector-to-target ratios (46) (Preprint).

### 3.2 Dismantling tumor immune evasion barriers

While enhancing tumor-intrinsic immunogenicity facilitates effector cell engagement, tumors maintain immune resistance through adaptive checkpoints, innate anti-phagocytic signals, and suppressive paracrine networks. Because KAT6A/B regulate the transcriptional programs underlying these immunosuppressive pathways, KAT6A/B targeting disrupts these defense mechanisms via chromatin remodeling, reversing immune tolerance within the TIME.

#### 3.2.1 Reversing programmed death-ligand 1 mediated T-cell exhaustion

Non-canonical metabolic-epigenetic modifications mediated by KAT6A drive CD274 (PD-L1) transcription. In a tumor microenvironment undergoing metabolic reprogramming or enriched with specific microbiota, the aberrant uptake of short-chain fatty acids expands the cellular propionyl-CoA pool. Functioning as a metabolic-transcriptional sensor, KAT6A utilizes this elevated propionyl-CoA to deposit histone H3 lysine 23 propionylation (H3K23pr) marks at the CD274 promoter (70). Compared to acetylation, propionylation increases steric hindrance and local hydrophobicity on the histone tail, weakening electrostatic histone–DNA interactions to promote open chromatin. This chromatin state recruits YEATS domain-containing or bromodomain-containing reader proteins, enriches positive transcription elongation factor b (P-TEFb), and facilitates RNA polymerase II (Pol II) pause-release to sustain efficient CD274 transcription (71).

Surface PD-L1 engagement with programmed cell death protein 1 (PD-1) on T cells dephosphorylates key T-cell receptor (TCR)-associated kinases within the immunological synapse, halting T-cell activation and inducing functional exhaustion (72–75). Clinical datasets demonstrate a positive correlation between KAT6A and PD-L1 expression. Importantly, both pharmacological inhibition using KAT6A-selective tools (e.g., WM-3835) and targeted epigenetic editing via CRISPR/dCas9 independently erase H3K23pr marks and silence CD274 transcription, providing complementary pharmacological and genetic evidence (47, 48). This releases T-cell suppression, restores granzyme B (GZMB) and perforin secretion, and reverses T-cell exhaustion.

Furthermore, KAT6A-mediated PD-L1 regulation is integrated into tumor spatial heterogeneity. Microenvironmental IL-6 drives distinct CD274 transcriptional programs across cellular subpopulations: non-stem cancer cells rely on the Janus kinase–signal transducer and activator of transcription 3 (JAK–STAT3)–FOS-like 1 (FRA1) axis, whereas cancer stem cells (CSCs) undergoing epithelial-to-mesenchymal transition (EMT) depend on zinc finger E-box binding homeobox 1 (ZEB1) activated via Src homology 2 domain-containing phosphatase 2 (SHP2)–phosphatidylinositol 3-kinase (PI3K)–protein kinase B (AKT) signaling (76). ZEB1 not only confers immune evasion properties to CSCs but also drives metastasis, establishing a core axis for primary resistance to ICB (77). KAT6A/B targeting is postulated to block the IL-6 cascade at its epigenetic origin in relevant contexts, potentially disrupting the STAT3-dependent pathway in non-stem cells and the ZEB1/EMT axis in CSCs—a hypothesis that requires direct experimental validation.

#### 3.2.2 Dismantling c-KIT-dependent paracrine suppressive networks

Stem-like subclones in solid tumors frequently overexpress the receptor tyrosine kinase c-KIT (CD117, encoded by KIT). Beyond driving intrinsic cell proliferation, c-KIT activation promotes the synthesis and secretion of immunosuppressive cytokines, including transforming growth factor-beta (TGF-β) and IL-10, through downstream kinase cascades (78, 79). Genetic KO/KD of KAT6A/B represses KIT transcription at its epigenetic origin by disrupting histone acetylation at the KIT promoter (49).

Downregulating cell-surface c-KIT attenuates downstream TGF-β and IL-10 paracrine signaling within the tumor microenvironment. Lower microenvironmental cytokine concentrations relieve local immune suppression, facilitating antigen-presenting cell (APC) maturation and restoring effector T-cell infiltration and cytotoxicity. Thus, KAT6A/B targeting reshapes the localized suppressive microenvironment by disrupting c-KIT-driven paracrine networks.

### 3.3 Reversing myeloid immunosuppression and reprogramming cytokine networks

Beyond regulating tumor-intrinsic evasion and adaptive immune suppression, KAT6A/B direct paracrine networks that establish an immunosuppressive myeloid compartment within the TIME. Targeting KAT6A/B alters cytokine transcription to functionally reprogram tumor-infiltrating myeloid cells, disrupting extrinsic myeloid barriers to restore antitumor immunity.

#### 3.3.1 Targeting the KAT6A–SMAD3 axis to reverse immunosuppressive myeloid reprogramming

KAT6A also shapes the myeloid compartment within the TIME by epigenetically regulating myeloid lineages. Mechanistically, KAT6A directly acetylates mothers against decapentaplegic homolog 3 (SMAD3) at lysine residues K20 and K117. This modification promotes SMAD3 binding to the co-activator tripartite motif-containing protein 24 (TRIM24) while sterically hindering interactions with the tumor suppressor TRIM33, enhancing SMAD3 transcriptional activity to drive the expression of immunomodulatory cytokines including IL-6, IL-22, and tumor necrosis factor-alpha (TNF-α) (50).

KAT6A–SMAD3-driven IL-6 paracrine signaling reprograms myeloid populations: tumor-associated macrophages (TAMs) upregulate CD163 to adopt an immunosuppressive phenotype, while myeloid-derived suppressor cells (MDSCs) accumulate within the tumor core (50, 80). In preclinical models, pharmacological inhibition of KAT6A using small-molecule tools (e.g., WM-1119) reduces MDSC recruitment by 60%–70%, repolarizes TAMs toward a pro-inflammatory antitumor phenotype, restores CD4^+^ and CD8^+^ T-cell infiltration, and inhibits metastasis (50). Reciprocally, MDSC-derived TGF-β activates tumor-intrinsic KAT6A–SMAD3 signaling, establishing a positive feedback loop that reinforces microenvironmental immune suppression.

#### 3.3.2 Senescence and the senescence-associated secretory phenotype

KAT6A/B inhibition induces stable cellular senescence in tumor cells, driving sustained cell-cycle arrest while reshaping the TIME through the SASP.

Mechanistically, catalytic inhibition of KAT6A reduces H3K9ac and H3K27ac levels at the promoters of target genes—including cell division cycle 6 (CDC6), enhancer of zeste homolog 2 (EZH2), and maternal embryonic leucine zipper kinase (MELK)—thereby derepressing the INK4A-ARF locus. Upregulation of p16INK4a and p19ARF works in concert with the p53–p21 axis to enforce G1 cell-cycle arrest. Pharmacological KAT6A/B blockade using small-molecule tools directly induces senescence markers in preclinical models, including elevated senescence-associated beta-galactosidase (SA-β-gal) activity (30, 81).

Senescent tumor cells secrete a spectrum of SASP factors—including IL-6, IL-8, and C-C motif chemokine ligand 2 (CCL2)—via NF-κB and cGAS–STING signaling (82). The immunological impact of the SASP is biphasic and time-dependent. Initially, early acute SASP factors recruit pro-inflammatory phagocytic macrophages, type 1 T helper (Th1) cells, and NK cells to clear senescent cells and neighboring tumor clones while increasing tumor antigenicity. With chronic exposure, however, persistent IL-6 and IL-8 recruit MDSCs and regulatory T (Treg) cells, while CCL2 chemoattracts C-C motif chemokine receptor 2-positive (CCR^2+^) myeloid cells to impair NK cell surveillance (83). Furthermore, sustained SASP signaling upregulates PD-L1 in residual cells via the JAK–STAT and NF-κB pathways, while IL-1α and TNF-α promote effector T-cell exhaustion (84).

This shift from acute immune surveillance to chronic immunosuppression indicates that the net immunological effect of KAT6A/B inhibitor-induced SASP depends heavily on exposure duration. This temporal dichotomy provides a conceptual framework for testing intermittent dosing schedules to capture initial immune-clearing benefits while avoiding chronic SASP-mediated immunosuppression—a hypothesis that requires rigorous PK/PD and *in vivo* experimental validation (85, 86).

### 3.4 Hypothesized pathways and future directions

The canonical mechanisms detailed in Sections 3.1–3.3 are empirically validated. The broader immunomodulatory footprint of KAT6A/B, however, extends into pathways where theoretical models currently outpace pharmacological data. Current literature links these acetyltransferases to CD47-mediated innate immune evasion, B7-H3-dependent lymphocyte exhaustion, splicing-derived neoantigen generation, and stromal/vascular remodeling. These associations remain largely inferred from the disruption of upstream genetic circuits (e.g., MYC, TGF-β/SMAD3, and VEGF signaling) rather than definitive small-molecule intervention. To maintain strict evidentiary boundaries, we classify these axes as working hypotheses.

#### 3.4.1 Blocking CD47-driven innate phagocytic escape

CD47 is a transmembrane protein that transmits a potent antiphagocytic signal by binding to signal regulatory protein α (SIRPα) on macrophages. Across solid tumors, elevated CD47 expression limits innate immune clearance by macrophages (87). Although direct pharmacological evidence linking KAT6A/B inhibition to CD47 downregulation is currently lacking, established epigenetic networks support an indirect inference in which KAT6A/B targeting could suppress CD47 transcription through dual potential mechanisms: cis-enhancer disruption and trans-factor depletion.

First, in solid tumors such as breast cancer, CD47 overexpression depends on a downstream super-enhancer (e.g., the E5 element). The open chromatin configuration of this region requires high levels of histone acetylation (e.g., H3K27ac), which recruits the epigenetic reader bromodomain-containing protein 4 (BRD4)—a key reader of the bromodomain and extra-terminal domain (BET) family—to assemble transcriptional complexes (88). Because KAT6A/B function as histone acetyltransferases, inhibiting their catalytic activity could reduce super-enhancer acetylation and impair BRD4 loading, mirroring the enhancer-disrupting actions of BET inhibitors.

Second, the oncogenic transcription factor MYC directly trans-activates CD47. Chromatin immunoprecipitation sequencing (ChIP-seq) demonstrates that MYC binds the CD47 promoter to drive its transcription (89). Because KAT6A/B activity maintains MYC transcription, KAT6A/B inhibition is predicted to deplete MYC protein levels, thereby decreasing trans-activation at the CD47 locus.

Although simultaneous suppression of cis-enhancers and trans-acting factors represents a mechanistically plausible model to disrupt the CD47–SIRPα axis, demonstrating that catalytic KAT6A/B inhibition reduces surface CD47 density sufficiently to trigger antibody-dependent cellular phagocytosis (ADCP) remains an unverified step. Rigorous flow-cytometric profiling and functional macrophage co-culture assays under small-molecule KAT6A/B inhibition will be essential to validate this inferential axis.

#### 3.4.2 Relieving B7-H3-mediated lymphocyte repression

In addition to paracrine factor networks, tumor cells suppress lymphocyte function through cell-surface checkpoints such as B7-H3 (CD276). Overexpressed across solid tumors—including neuroblastoma and prostate cancer—B7-H3 directly inhibits NK cell and effector T-cell cytotoxicity (90). B7-H3 expression relies heavily on MYC, which directly activates CD276 transcription and represses the microRNA-29 (miR-29) family (microRNAs targeting CD276 mRNA for degradation), thereby sustaining B7-H3 protein levels (90, 91).

Because MYC remains challenging to target directly with small molecules, inhibiting upstream epigenetic machinery that sustains MYC transcription offers an alternative therapeutic strategy. KAT6A deposits H3K9ac at target gene promoters, recruiting the epigenetic reader eleven-nineteen leukemia protein (ENL) and assembling the super elongation complex (SEC) to release paused Pol II and drive transcriptional elongation of oncogenes including MYC (92). Consequently, genetic depletion of KAT6A disrupts MYC-dependent transcriptional programs and prolongs survival in lymphoma models (30). By depriving MYC of epigenetic support, KAT6A/B depletion or degradation is postulated to indirectly downregulate B7-H3 and restore miR-29 expression. Nevertheless, inferring that catalytic KAT6A/B inhibition lowers cell-surface B7-H3 density sufficiently to reactivate lymphocyte cytotoxicity remains an unverified step. Confirming changes in KAT6A/B occupancy and chromatin accessibility at the CD276 locus alongside functional NK/T-cell cytotoxicity assays under small-molecule inhibition will be essential to validate this inferential axis.

#### 3.4.3 Antigen repertoire expansion through transcription–splicing decoupling

RNA polymerase II (Pol II) elongation velocity governs co-transcriptional splicing kinetics (93). Histone acetylation deposited by KAT6A/B, particularly H3K23ac, supports Pol II processivity and elongation across active gene bodies (94). Consequently, genetic depletion or catalytic inhibition of KAT6A/B can induce Pol II stalling, compromising co-transcriptional splicing fidelity and triggering aberrant splicing events, including intron retention, exon skipping, or cryptic splice site selection.

Analogous to DNA methyltransferase (DNMT) inhibitors that generate immunogenic peptides through endogenous retrovirus (ERV) derepression or altered splicing (95, 96), KAT6A/B inhibition-induced splicing infidelity is postulated to yield frameshifted or non-canonical protein products. When presented on MHC-I, these aberrant peptides may function as novel neoantigens (96). However, translating this epigenetic–splicing linkage into functional anti-tumor immunity remains a hypothetical concept. Direct evidence demonstrating that small-molecule KAT6A/B inhibitors alter the immunopeptidome sufficiently to elicit T-cell reactivity is currently lacking. High-throughput mass spectrometry-based neoepitope profiling and functional T-cell activation assays under catalytic KAT6A/B inhibition will be imperative to test this working hypothesis.

#### 3.4.4 Stromal and vascular microenvironment modulation

Cancer-associated fibroblasts (CAFs) and the dense extracellular matrix (ECM) networks they secrete constitute the primary physical barrier impeding the infiltration of CD8^+^ T cells into the tumor core. CAF hyperactivation and collagen cross-linking depend on sustained transcriptional output through the canonical TGF-β/SMAD3 signaling pathway (97). Given that KAT6A acts as a co-activator to directly drive the epigenetic modification and transcriptional activity of SMAD3 (50), genetic depletion of KAT6A would be expected to attenuate SMAD3-dependent pro-fibrotic transcription, reducing expression of α-smooth muscle actin (α-SMA), collagen I, and fibronectin. Consequently, targeting KAT6A is postulated to promote CAF deactivation and reduce tissue stiffness. However, establishing that small-molecule KAT6A/B catalytic inhibitors effectively deactivate CAFs and soften the dense ECM *in vivo* remains an unverified inference; validation requires 3D organoid-stromal co-cultures and tissue biomechanics assays under catalytic inhibition.

Concurrently, aberrant tumor neovasculature elevates interstitial fluid pressure and induces endothelial anergy—partially via downregulation of adhesion molecules, including intercellular adhesion molecule-1 (ICAM-1) and vascular cell adhesion molecule-1 (VCAM-1)—restricting transendothelial lymphocyte extravasation (98). Transcription of pro-angiogenic factors, such as vascular endothelial growth factor A (VEGFA), relies on hypoxia-responsive transcription factors, including signal transducer and activator of transcription 3 (STAT3) and hypoxia-inducible factor 1-alpha (HIF-1α). KAT6A maintains open chromatin at these transcriptional loci across multiple tumor types. Consequently, KAT6A inhibition suppresses pro-angiogenic factor expression, pruning pathological microvessels and promoting vascular normalization by attenuating vascular endothelial growth factor receptor 2 (VEGFR2) signaling, upregulating angiopoietin-1, and recruiting pericytes (99, 100). While these genetic data provide a theoretical rationale for vascular normalization, attributing functional microvascular pruning, pericyte recruitment, or restored lymphocyte trafficking directly to small-molecule KAT6A/B inhibition remains a hypothesis awaiting *in vivo* microcirculatory flow evaluation.

Translating vascular remodeling into clinical strategies is further complicated by isoform-specific functional divergence. Beyond endothelial vessels, tumors can undergo vasculogenic mimicry (VM)—fluid-conducting channels formed by transdifferentiated tumor cells independent of endothelial cells (101). KAT6A and KAT6B display functional antagonism in this context. While KAT6A supports pro-angiogenic programs, KAT6B suppresses VM formation. In VM-rich tumors, the transcription factor zinc finger protein 384 (ZNF384) drives channel formation; KAT6B acetylates ZNF384 to promote its ubiquitin–proteasome-mediated degradation. Conversely, when KAT6B is silenced via small nucleolar RNA 17 (SNORD17)-mediated hypermethylation, ZNF384 accumulates and drives dense VM network assembly (102).

This functional antagonism presents a significant challenge for drug design: non-selective pan-KAT6A/B inhibitors, while disrupting classical endothelial vessels, may concurrently induce compensatory VM through KAT6B inhibition. Evaluating whether KAT6A-selective inhibitors achieve physiological vascular normalization without triggering KAT6B-dependent VM escape represents an essential milestone for future translational studies.

### 3.5 Dissecting the dual-compartment tension and systemic costs of immune intervention: a hypothesis-generating framework

Balancing local antitumor activity against systemic toxicity remains a major challenge in KAT6A/B inhibition. We present a conceptual framework centered on this dual-compartment conflict. Rather than a simple spatial divide between local activation and systemic suppression, systemic KAT6A/B blockade directly drives the attrition of immune clones. Meanwhile, inside the TIME, targeted blockade triggers compensatory escape—notably through erb-b2 receptor tyrosine kinase 2 (ERBB2) upregulation and ferroptosis-driven lipid peroxidation that injures bystander T cells. Mapping these local and systemic limits explains why time-dependent strategies, such as intermittent dosing schedules, are required to improve therapeutic outcomes (**Figure 2**).

**Figure 2:**
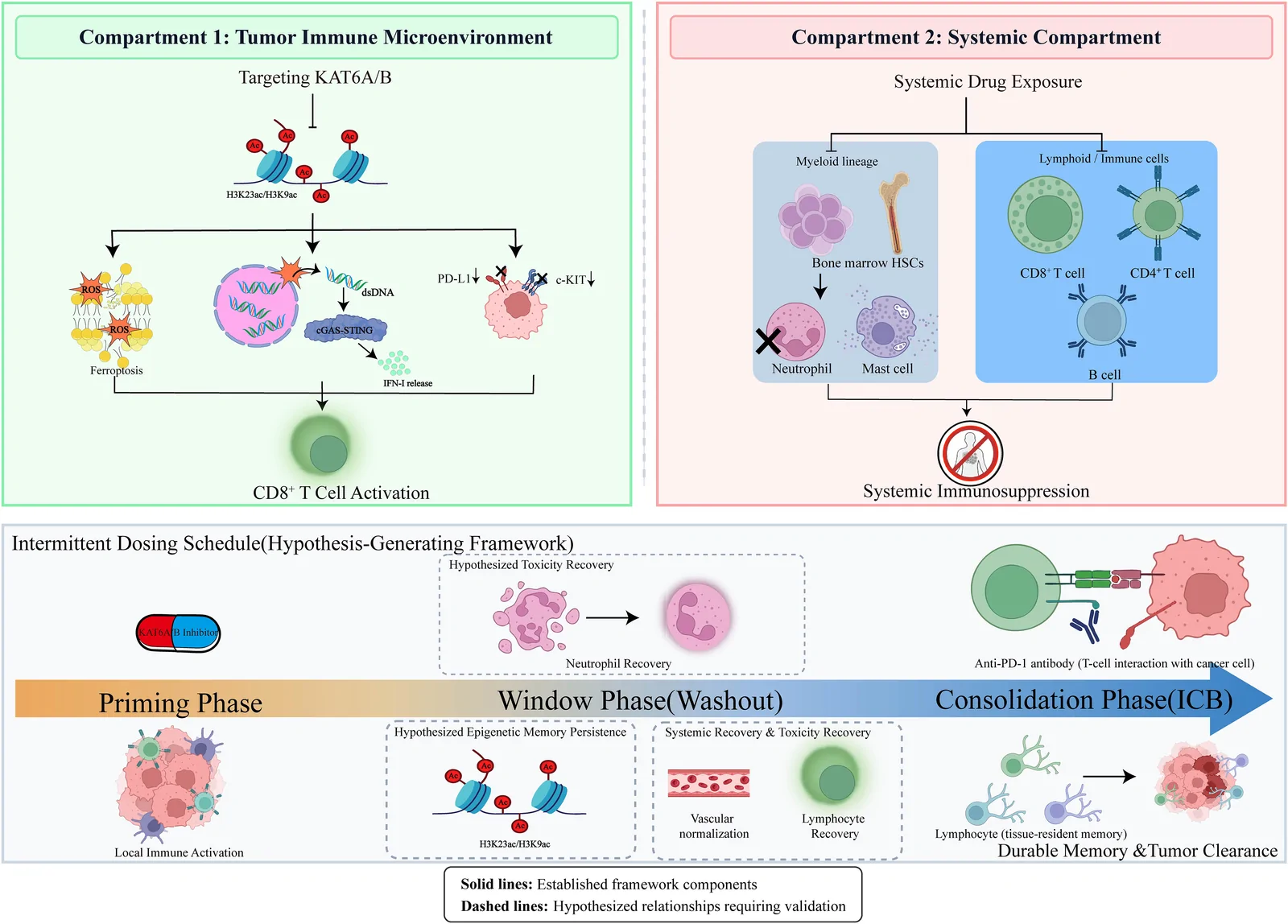
Dual-compartment hypothesis and intermittent dosing strategy for KAT6A/B inhibition. Compartment 1: tumor immune microenvironment: Targeting KAT6A/B depletes H3K23ac/H3K9ac to trigger ferroptosis, cGAS–STING-mediated IFN-I release, and downregulation of immune checkpoints (PD-L1, c-KIT), culminating in local CD8^+^ T-cell activation. Compartment 2: systemic compartment: Prolonged systemic drug exposure suppresses bone marrow HSCs, leading to neutropenia and systemic immunosuppression across myeloid and lymphoid lineages. Intermittent dosing schedule (hypothesis-generating framework): A three-phase regimen designed to balance local antitumor efficacy against systemic toxicity. Priming Phase: Initial KAT6A/B inhibition establishes local immune activation within the TIME. Window Phase (Washout): Drug holiday allows systemic hematological recovery (neutrophil/lymphocyte rebound) and vascular normalization while maintaining persistent epigenetic memory in tumor tissues. Consolidation Phase (ICB): Subsequent immune checkpoint blockade (e.g., anti-PD-1) capitalizes on the primed immune microenvironment to achieve durable T-cell memory and tumor clearance. Solid lines indicate established framework components; dashed lines indicate hypothesized relationships requiring further validation. Created with BioRender.com.

#### 3.5.1 Systemic hematopoietic suppression and immune clonal attrition

KAT6A regulates lymphocyte lineage commitment and effector functions, meaning its inhibition can trigger immune clonal attrition. During B-cell development, KAT6A binds the transcription start sites of key regulatory genes, including Meis1, to maintain expression of Pbx1 and the Hoxa gene cluster, sustaining progenitor B-cell proliferation. Disrupting KAT6A dismantles this transcriptional program, causing B-cell developmental arrest and clonal depletion (55). In CD8^+^ T cells, KAT6A deposits H3K9ac at regulatory elements within the cluster of differentiation 8 (Cd8) locus (e.g., the E8_i_ enhancer) to keep chromatin accessible; loss of KAT6A impairs the generation of killer cell lectin-like receptor G1-positive, interleukin-7 receptor subunit alpha-negative (KLRG-1^+^CD127^-^) effector memory T cells, blunting CD8^+^ T-cell cytotoxicity (103). In CD4^+^ T cells, KAT6A bridges metabolic reprogramming with inflammatory differentiation by acetylating promoter regions of key glycolytic genes—hexokinase 2 (Hk2), pyruvate kinase M (Pkm), and lactate dehydrogenase A (Ldha)—which recruits HIF-1α and MYC. Blockade of this metabolic driver with the small-molecule inhibitor WM-1119 induces T-cell anergy (56).In innate immune cells and myeloid lineages, KAT6A/B inhibition exerts epigenetic suppressive effects that translate into on-target hematopoietic toxicity. KAT6A inhibition reduces H3K14ac and H3K27ac levels, dampening mitogen-activated protein kinase (MAPK)/NF-κB signaling and activator protein 1 (AP-1) activity. This suppresses mast cell degranulation and cytokine release (IL-4 and TNF-α) (104), while KAT6A depletion simultaneously compromising pro-inflammatory macrophage polarization (105). Systemically, bone marrow suppression limits the therapeutic window of KAT6A/B inhibitors. In a Phase I trial evaluating PF-07248144 in ER^+^/HER2^-^ metastatic breast cancer, neutropenia was the primary dose-limiting toxicity, occurring in approximately 48% of patients (grade 3 or higher in 37.2%) (15). High rates of severe myelosuppression underscore the essential role of KAT6A in normal hematopoiesis and constrain continuous dosing regimens. Managing systemic hematopoietic toxicity through intermittent schedules or isoform-selective targeting will be critical to optimizing KAT6A/B-based combination immunotherapies.

#### 3.5.2 Local immune escape driven by compensatory ERBB2 activation

In ER^+^/HER2^+^ breast cancer, KAT6A/B links endocrine receptor signaling to the TIME. At the estrogen receptor 1 (ESR1) promoter, KAT6A/B recruits the Menin–lysine methyltransferase 2A (KMT2A) complex to maintain an open chromatin configuration; small-molecule inhibition of KAT6A/B (e.g., via PF-9363) depletes local histone modifications and downregulates estrogen receptor alpha (ERα) expression (52). ERα functions not only as a pro-proliferative driver but also as a trans-repressor of ERBB2 by binding intronic cis-regulatory elements and recruiting paired box 2 (PAX2), which competitively excludes the co-activator amplified in breast cancer 1/steroid receptor coactivator 3 (AIB-1/SRC-3). Consequently, KAT6A/B inhibition or endocrine resistance relieves PAX2-mediated repression, enabling SRC-3 recruitment and triggering compensatory ERBB2 activation (51).

This derepression-driven ERBB2 hyperactivation exerts biphasic effects on the TIME. Initially, ERBB2-induced genomic instability and micronucleus formation can transiently recruit effector T cells via IFN-I signaling (106). However, sustained ERBB2 signaling ultimately drives immune evasion by downregulating MHC-I to impair antigen presentation (107), upregulating PD-L1, and recruiting MDSCs via JAK–STAT3 and NF-κB activation (108). In the context of systemic myelosuppression and reduced peripheral T-cell supply, ERBB2-mediated microenvironmental deterioration accelerates the loss of remaining intratumoral T cells, driving them toward terminal exhaustion.

#### 3.5.3 T cell bystander injury and terminal exhaustion driven by ferroptosis-associated lipid toxicity

Although targeted therapy-induced ferroptosis can promote a more inflamed TIME through DAMP release, this process induces bystander lipid toxicity. Ferroptotic stress can deplete lipid-dependent immunosuppressive cells, such as Treg cells and immunosuppressive macrophages, alleviating local immune tolerance. However, lipid peroxidation concurrently causes bystander injury to DCs and NK cells, impairing antigen presentation and early cytotoxic cascades (59).

Inside the TIME, accumulating lipid ROS subject infiltrating CD8^+^ T cells to intense metabolic strain, accelerating their trajectory toward terminal exhaustion. Mechanistically, CD8^+^ T cells upregulate CD36 to import oxidized lipids, such as oxidized low-density lipoprotein (oxLDL). This influx elevates intracellular polyunsaturated fatty acids, triggering p38 mitogen-activated protein kinase (p38 MAPK) signaling while blunting IFN-γ production. Concurrently, lipid toxicity impairs mitochondrial function: activation of Ac-CoA carboxylase (ACC) generates malonyl-CoA, which allosterically inhibits CPT1A and suppresses fatty acid oxidation (FAO), compromising effector T-cell bioenergetics (54). At the transcriptional and epigenetic levels, high lipid stress induces endoplasmic reticulum stress that activates the IRE1α–XBP1 axis to drive PDCD1 (encoding PD-1) expression. Lipid peroxidation also alters global chromatin accessibility, sustaining high expression of the transcription factor thymocyte selection-associated high mobility group box (TOX) and establishing a terminal exhaustion phenotype characterized by T-cell immunoglobulin and mucin-domain containing-3 (TIM-3) and lymphocyte-activation gene 3 (LAG-3) co-expression (54, 109). Resolving these spatiotemporal dynamics will require single-cell and spatial multi-omics approaches.

It should be emphasized that this dual-compartment framework is heuristic and hypothesis-generating; it does not provide quantitative compartment modeling, and the term “compartment” is used to organize local versus systemic effects rather than as a formal pharmacokinetic entity. Key operational parameters—including epigenetic memory half-life, H3K23ac turnover, immune subset heterogeneity, and nonlinear dose–response relationships—remain unresolved and warrant future quantitative PK/PD modeling and experimental validation.

## 4 Clinical translation

Although KAT6A/B inhibitors have been reported to remodel the TIME in preclinical models, clinical translation requires converting local intratumoral immune activation into durable survival benefits while mitigating systemic hematopoietic toxicity. Several KAT6A/B-targeted agents have advanced to early-phase clinical evaluation. Preliminary trial data confirm target druggability and demonstrate robust target engagement; however, direct clinical validation of the dual-compartment hypothesis remains an ongoing objective. Specifically, H3K23ac suppression serves as a validated pharmacodynamic (PD) biomarker of direct target engagement, whereas dose-limiting neutropenia highlights the clinical challenge of systemic hematopoietic suppression in the secondary compartment.

### 4.1 Clinical validation of KAT6A/B inhibition and toxicity

PF-07248144, a pan-KAT6A/B catalytic inhibitor, was the first in its class to enter clinical evaluation. Preliminary PD analyses from early-phase disclosures demonstrated median H3K23ac inhibition of at least 70% in patient peripheral blood mononuclear cells (PBMCs) across evaluated doses, reaching up to 97% in paired tumor biopsy tissues (15, 35). These findings support H3K23ac suppression as a PD biomarker for KAT6A/B target occupancy in human clinical samples, providing proof-of-mechanism without directly inferring downstream clinical efficacy (110).

Safety data identified neutropenia as the primary DLT of PF-07248144, reflecting the role of KAT6A in normal myeloid hematopoiesis and illustrating the clinical challenge of systemic myelosuppression during catalytic KAT6A/B inhibition (15, 36). To mitigate on-target hematological toxicity, the selective PROTAC degrader PRT13722 was developed. Preclinical company disclosures report that PRT13722 achieves nanomolar degradation of KAT6A while sparing KAT6B. In colony-forming unit-granulocyte macrophage (CFU-GM) progenitor assays, PRT13722 was reported to exhibit minimal myelosuppressive toxicity, in contrast to pan-KAT6A/B catalytic inhibitors (41). Isoform-selective degradation may therefore provide a medicinal chemistry strategy to uncouple systemic hematopoietic toxicity from localized antitumor mechanisms, although its therapeutic window and proposed immune-modulatory effects require further clinical validation.

### 4.2 Clinical response patterns and future validation of microenvironmental remodeling

In heavily pretreated patients with ER^+^/HER2^-^ metastatic breast cancer, PF-07248144 combined with fulvestrant achieved an objective response rate (ORR) of 30.2% and a clinical benefit rate (CBR) of 51.2%, with consistent efficacy across treatment lines and biomarker subgroups (including ESR1 and PIK3CA/AKT1/PTEN alterations) (15). However, these clinical outcomes represent composite efficacy endpoints that cannot disentangle tumor cell-autonomous antiproliferative effects, endocrine resensitization, and potential non-cell-autonomous immunomodulation. While this driver mutation–independent activity reflects broad epigenetic activity, directly attributing observed clinical responses to TIME remodeling lacks definitive clinical proof.

Preclinical studies suggest that KAT6A/B inhibition suppresses PD-L1 expression and may downregulate additional checkpoints and paracrine signals. Nevertheless, these proposed immune PD mechanisms have not been directly measured in human patient samples. Consequently, whether KAT6A/B blockade promotes functional TIME remodeling in patients remains a testable hypothesis rather than a clinically established mechanism.

To validate the immune-remodeling hypothesis in clinical settings, prospective trials must incorporate comprehensive translational correlative studies. Specifically, confirming KAT6A/B-mediated TIME engagement requires paired pre- and post-treatment tumor biopsies evaluated through multiplex immunohistochemistry (mIHC), spatial transcriptomics (ST), and single-cell RNA sequencing (scRNA-seq) to quantify cytotoxic T-cell infiltration and myeloid repolarization, integrated with circulating cytokine profiling to monitor systemic immune dynamics.

### 4.3 Immunomodulatory potential of selective and dual-target inhibitors

To mitigate hematological toxicities associated with catalytic KAT6A/B inhibition and overcome adaptive resistance, next-generation selective and multi-target inhibitors are advancing through clinical evaluation via two distinct strategies: refinement of isoform-selective KAT6A/B inhibitors and dual KAT6/7 targeting to overcome epigenetic redundancy.

OP-3136 is a selective small-molecule catalytic inhibitor of KAT6A/B with nanomolar potency (94). Preliminary conference data suggest that OP-3136 induces single-agent tumor regression in ovarian cancer (OVCAR3; tumor growth inhibition [TGI] rate of 104.5%–105.4%) and non-small cell lung cancer (NSCLC; LCLC-97TM1) models, and may demonstrate synergism when combined with endocrine therapies or cyclin-dependent kinase 4/6 (CDK4/6) inhibitors. Its isoform-selective profile is proposed to minimize interference with systemic hematopoiesis, potentially providing a safer biochemical foundation for combinations with ICB. Phase I testing of OP-3136 is currently evaluating its safety and preliminary efficacy in patients with advanced solid tumors (16).

To address epigenetic redundancy, dual KAT6/7 inhibition has emerged as a distinct translational approach. According to company disclosures, IDE574 is a selective KAT6/7 dual inhibitor that targets KAT6A/B and KAT7 while sparing other MYST family acetyltransferases. Because KAT6A/B and KAT7 cooperatively regulate chromatin accessibility and cell identity, tumors frequently hijack both enzymes to sustain lineage plasticity and survival dependencies. Dual KAT6/7 blockade is proposed to prevent transcriptional compensation and may dismantle lineage maintenance programs. Company disclosures indicate that IDE574 induces deep tumor regression in patient-derived xenograft (PDX) models harboring 8p11 chromosomal amplifications (present in approximately 15% of breast cancers and 17.5% of squamous NSCLC) or displaying lineage dependencies. An Investigational New Drug (IND) application for IDE574 was submitted in December 2025, and a Phase 1 trial has since been initiated in patients with 8p11-amplified malignancies (111).

Similarly, according to preliminary company disclosures, KC1086 is a potent, nanomolar KAT6/7 dual inhibitor. Isolated KAT6A/B blockade can induce compensatory recruitment of the structurally homologous KAT7, driving transcriptional takeover that reactivates the cell cycle and re-establishes an immunosuppressive microenvironment. Company disclosures report that dual KAT6/7 inhibition with KC1086 prevented this adaptive feedback and yielded over 90% tumor growth inhibition as a single agent in ER^+^/HER2^-^ breast cancer cell line-derived xenograft (CDX) models. KC1086 is reported to exhibit high oral bioavailability, a pharmacokinetic profile supporting once-daily dosing, and a favorable safety margin (112). Clinical evaluation began following regulatory approvals from the National Medical Products Administration (NMPA) and US FDA, with Phase I trials currently underway.

By blocking epigenetic redundancy, dual KAT6/7 targeting may help dismantle lineage maintenance and immune escape programs at their source, potentially representing a therapeutic strategy to overcome microenvironment-mediated resistance following isolated KAT6A/B inhibition, although this concept remains to be validated in clinical settings.

### 4.4 Clinical combination strategies based on mechanistic synergy

Monotherapy with KAT6A/B inhibitors is constrained by adaptive resistance. Under therapeutic pressure, tumor cells sustain oncogenic transcriptional programs by recruiting paralogous acetyltransferases (e.g., KAT7) or alternative transcriptional co-activators (32). Overcoming these compensatory escape mechanisms requires rationally designed combination strategies, particularly integrating KAT6A/B blockade with endocrine therapies and CDK4/6 inhibitors.

#### 4.4.1 Combination with endocrine therapy to overcome acquired resistance

KAT6A/B inhibition deprives ESR1 of epigenetic support, concurrently attenuating ERα-driven proliferative signaling and relieving PAX2-mediated repression of ERBB2 (Section 3.5.2), providing a strong mechanistic rationale for co-targeting endocrine signaling (32). Combining KAT6A/B inhibitors with selective estrogen receptor degraders (SERDs, e.g., fulvestrant) achieves dual-level suppression: KAT6A/B inhibition (e.g., via PF-07248144) shuts down ESR1 transcription, whereas SERDs degrade synthesized ERα protein. This concurrent targeting of transcriptional output and protein stability bypasses mutational resistance mechanisms that limit receptor-targeted monotherapies. Preliminary conference data indicate that this combination induced circulating tumor DNA (ctDNA) clearance in both ESR1-mutant and wild-type clones, with reported objective response rates (ORRs) of 33.3% and 27.8%, respectively. Dose escalation from 1 mg to 5 mg was reported to produce parallel increases in ORR and median duration of response (DOR) (15, 113).

Next-generation strategies are transitioning from catalytic inhibition to targeted degradation. For example, preliminary conference data suggest that ISM5043 downregulates ERα protein abundance to synergize with endocrine agents (38). Similarly, company disclosures report that the selective KAT6A degrader PRT13722 depletes KAT6A protein, eliminating both its catalytic activity and scaffold functions to induce durable tumor regression in models of profound endocrine resistance (114). Additionally, co-targeting KAT6A/B and the Menin complex represents a complementary epigenetic strategy to overcome refractory endocrine resistance (52).

#### 4.4.2 Synthetic lethal effects of combination with CDK4/6 inhibitors

CDK4/6 inhibitors combined with endocrine therapy constitute the standard frontline regimen for HR^+^/HER2^-^ advanced breast cancer; however, acquired resistance driven by cell-cycle reprogramming frequently develops. Following CDK4/6 inhibitor progression, fulvestrant monotherapy provides limited efficacy, with a clinical benefit rate (CBR) of 13.7% and a median progression-free survival (PFS) of approximately 2 months (15). Combining KAT6A/B inhibitors with CDK4/6 inhibitors targets a synthetic lethal vulnerability: KAT6A regulates ESR1 transcription via H3K23ac while also supporting the expression of core components within the CDK4/6–Rb–E2F signaling pathway (32). KAT6A/B inhibition reduces *de novo* transcription of key cell-cycle drivers (e.g., CCND1), whereas CDK4/6 inhibitors block kinase activity at the protein level. Co-targeting both nodes synergistically suppresses Rb phosphorylation, dismantling E2F-driven transcriptional programs.

Preclinical studies support the therapeutic rationale of this combination. Preliminary conference data suggest that a triplet regimen combining OP-3136, the CDK4/6 inhibitor ribociclib, and anti-estrogen therapy induced marked tumor regression in multiple models (115). Similarly, company disclosures report that KC1086 combined with palbociclib achieved a TGI rate of 101% in ZR-75–1 xenografts (112), suggesting synergistic antitumor activity upon dual KAT6A/B and CDK4/6 blockade (116).

In clinical evaluation, the Phase I trial of PF-07248144 (NCT04606446) enrolled patients with prior CDK4/6 inhibitor exposure in its dose-expansion cohort (113). Preliminary conference data indicate that clinical responses in this pretreated population suggest KAT6A/B inhibitors may maintain therapeutic efficacy following CDK4/6 inhibitor resistance. Exploratory pharmacodynamic analyses reported that PF-07248144 downregulates ER signaling and suppresses KAT6A/B-dependent MYC transcriptional programs, accompanied by clearance of resistant clones in circulating tumor DNA (ctDNA) (117).

Clinical development is evaluating these combinations across treatment settings. According to clinical trial registry entries, a global Phase III trial (NCT07062965; VERITAC-3) is comparing PF-07248144 plus fulvestrant head-to-head against everolimus in patients pretreated with CDK4/6 inhibitors (118). In the frontline setting, preliminary conference data indicate that a Phase I cohort is evaluating a triplet combination of PF-07248144, fulvestrant, and palbociclib to assess concurrent epigenetic and cell-cycle inhibition (113). Preliminary conference data further suggest that genomic alterations, such as KAT6A amplification, are being investigated as potential predictive biomarkers to select patient subsets candidate for triplet regimens (119).

### 4.5 Exploring intermittent intervention strategies based on immune kinetics

To balance local intratumoral immune activation against systemic immunosuppression, a kinetics-guided intervention—termed transient epigenetic priming—offers a testable alternative requiring empirical validation (120) (**Figure 2**). This strategy operates sequentially: short-course, high-dose KAT6A/B inhibition rapidly induces tumor ferroptosis and cGAS–STING pathway activation, promoting a more inflamed TIME (priming phase) (121); a subsequent drug washout period then alleviates continuous systemic lymphocyte suppression, allowing newly recruited T cells to undergo effector differentiation and infiltrate the tumor core without epigenetic inhibition (122). Finally, in the consolidation phase, immune checkpoint inhibitors (e.g., anti-PD-1 antibodies) are administered during the washout window to capitalize on the pre-conditioned, receptive TIME (18).

Executing this three-phase approach hinges on uncoupling three interconnected kinetic variables. First, local immune activation kinetics dictates whether chromatin remodeling, genomic stress, and danger-signal release can trigger innate immune sensing before systemic toxicities emerge (123). Second, the adaptive immune system’s recovery half-life governs safety; peripheral lymphopoiesis and CD4^+^ T-cell metabolic recovery determine the window for introducing subsequent immunotherapies (124). Third, the persistence of intratumoral chemokine gradients during washout defines how effectively epigenetic memory bridges priming to consolidation (125).

Optimizing intermittent schedules thus requires balancing two opposing thresholds: the priming duration must surpass the threshold for local immune activation, whereas the washout interval must allow systemic hematopoietic recovery without outlasting the decay rate of intratumoral epigenetic memory. Although exact kinetic parameters require empirical clinical validation, this framework provides a theoretical base for quantitative dosing schedule optimization via systems pharmacology modeling (126).

Translating this strategy into clinical practice demands integrated pharmacodynamic biomarkers reflecting net immunologic benefit. A dual-signal monitoring strategy could track intratumoral CD8^+^ T-cell infiltration or serum CXCL10 levels to gauge local activation (127), while monitoring absolute neutrophil counts or peripheral blood mononuclear cell (PBMC) H3K23ac suppression to monitor systemic toxicity. The ratio of intratumoral H3K23ac suppression to peripheral neutrophil nadir during washout may serve as a quantitative PK/PD metric for therapeutic window optimization.

Validating the intermittent dosing hypothesis will require systematic preclinical and translational investigations. Establishing that local immune activation precedes systemic immunosuppression necessitates detailed kinetic profiling via serial tumor and blood sampling following KAT6A/B inhibition to resolve the onset of intratumoral T-cell infiltration relative to peripheral cytopenia. Quantitative PK/PD modeling in immunocompetent tumor models can establish dose-time relationships for local activation versus systemic toxicity (126). Furthermore, head-to-head comparisons of intermittent versus continuous schedules are essential to confirm whether a defined “window” expands the therapeutic index and enhances subsequent immune checkpoint blockade—a strategy already being explored in other epigenetic classes, such as KDM4 inhibitors (128). Finally, longitudinal immune monitoring—tracking peripheral lymphocyte counts, T-cell activation markers, and intratumoral infiltrates—will help pinpoint the optimal kinetic window for first-in-human intermittent dosing trials (129).

## 5 Core challenges in clinical translation

Despite promising preclinical and early clinical data for KAT6A/B-targeted agents and combination regimens, several obstacles hinder the broader clinical adoption of these agents. These translational challenges fall into three interconnected categories: on-target hematopoietic toxicity within normal stem cell compartments, marked context-dependency of therapeutic efficacy across diverse genetic backgrounds, and multifaceted resistance mechanisms driven by non-catalytic scaffolding functions coupled with epigenetic plasticity. Critically, these translational hurdles are appraised herein within a conceptual, hypothesis-generating framework—particularly regarding dual-compartment dynamics and optimized dosing schedules—that awaits rigorous prospective experimental and clinical validation.

### 5.1 Target-intrinsic multi-system toxicity risks

During cardiac development, KAT6A catalyzes H3K9ac deposition in mesodermal tissue to directly activate transcription of Tbx1 and Tbx5, driving morphogenesis of the cardiac outflow tract, aortic arch, and ventricular septum. Mesodermal Kat6a deletion downregulates Tbx1 and Tbx5 expression, causing high-penetrance ventricular septal defects (VSDs) and aortic arch malformations, and thymic hypoplasia reminiscent of DiGeorge syndrome (130). Critically, these severe structural malformations reflect developmental genetic phenotypes resulting from embryonic gene KO, which must not be directly extrapolated to adult pharmacological inhibition. Because adult tissue homeostasis lacks embryonic tissue remodeling, whether short-term pharmacological catalytic blockade or protein degradation in adult patients induces comparable cardiotoxicity remains to be systematically validated in adult preclinical models. Nevertheless, these developmental roles necessitate formal evaluations of teratogenic and reproductive toxicities, particularly when treating patients of childbearing potential.

Beyond its acetyltransferase catalytic activity, KAT6A exerts crucial “non-catalytic scaffold functions” mediated by its N-terminal domain. Evidence demonstrates that the depletion of hematopoietic stem cells (HSCs) resulting from complete KAT6A KO (protein loss) is markedly more severe than that caused by the mere loss of catalytic activity (131). Mechanistically, KAT6A physically recruits and stabilizes transcription factors—including the MLL complex, RUNX1, and PU.1—to co-activate core hematopoietic genes, including HOXA5, HOXA7, and HOXA9. Disruption of KAT6A protein integrity or its protein–protein interaction (PPI) network impairs HSC self-renewal, manifesting clinically as neutropenia and lymphopoiesis defects (132). Conversely, KAT6A gene amplification drives hematologic malignancies such as AML (7). Consequently, targeted protein degraders—such as PRT13722, a selective KAT6A degrader with an IND filing planned for mid-2026 (company disclosure/preliminary data)—or PPI disrupters that dismantle this structural scaffold carry a higher risk of severe myelosuppression compared to isoform-selective catalytic inhibitors that preserve the KAT6A protein scaffold.

Beyond systemic myelosuppression, target-mediated toxicity also manifests locally within the TIME. Although KAT6A/B inhibition induces cancer cell death via ferroptosis, the concomitant release of toxic oxidized lipids causes bystander injury to tumor-infiltrating T lymphocytes, NK cells, and DCs. This local immunosuppressive secondary toxicity further compresses the narrow therapeutic window, emphasizing the need for next-generation modalities that carefully balance catalytic suppression, scaffold integrity, and local immune persistence.

### 5.2 Context-dependent functional heterogeneity of KAT6A/B

The antitumor efficacy of KAT6A/B inhibition is context-dependent, shaped by functional divergence between family members and specific tumor genomic backgrounds.

Although KAT6A/B generally function as oncogenic drivers in solid tumors, KAT6B displays tumor-suppressive roles in specific cancer types. In SCLC, KAT6B catalyzes H3K23ac to maintain open chromatin and active transcription of a tumor-suppressive network containing BRM and CDH1 (encoding E-cadherin). High-frequency loss or deletion of KAT6B in SCLC causes chromatin condensation and epigenetic silencing of this pathway, accelerating tumor growth and hepatic metastasis (133). Similarly, in neuroblastoma, germline or somatic missense variants in KAT6B disrupt epigenetic homeostasis through dominant-negative mechanisms to increase cancer susceptibility (134). This context-dependent heterogeneity warns that non-selective pan-KAT6A/B inhibitors risk inadvertently suppressing KAT6B’s tumor-suppressive activity, potentially accelerating disease progression in KAT6B-deficient or -dependent tumor contexts.

The functional output of KAT6A is similarly governed by genomic background. While KAT6A/B inhibition can trigger cellular senescence in specific contexts, KAT6A exerts an opposing, tumor-suppressive function in TP53 wild-type tumors reliant on the p53–p21 signaling axis. As a transcriptional co-activator of p53, KAT6A acetylates p53 at Lys120 and Lys382 within promyelocytic leukemia (PML) nuclear bodies to activate CDKN1A (encoding p21) transcription, promoting senescence (135). Crucially, the evidence establishing this tumor-suppressive role relies predominantly on genetic KO/KD models. This distinction reveals a critical pharmacological divergence between catalytic inhibition and protein degradation. In TP53 wild-type tumors, catalytic inhibitors that selectively block acetyltransferase activity while retaining the KAT6A protein scaffold may present a safer therapeutic profile, as the preserved structural scaffold can continue acting as a co-activator to sustain baseline p53-mediated tumor-suppressive pressure. Conversely, PROTAC degraders or complete genetic depletion completely eliminate the KAT6A protein, risking the full collapse of p53 co-activation, impairing senescence, and inadvertently conferring a survival advantage to tumor cells. This functional duality highlights the necessity of biomarker-guided patient selection, incorporating TP53 mutation status and KAT6B copy-number profiling in clinical trial designs.

### 5.3 Multidimensional mechanisms of acquired resistance

Acquired resistance to KAT6A/B-targeted therapies arises through two primary mechanisms: metabolic reprogramming that drives substrate-level target competition and non-catalytic functional compensation by epigenetic plasticity.

Most clinical-stage KAT6A/B inhibitors, including PF-07248144, function as Ac-CoA competitive inhibitors. Tumor cells can establish substrate-level resistance through metabolic rewiring. In AML and multiple myeloma, tumor cells bypass negative feedback within the CoA biosynthetic pathway through genetic alterations, such as PANK3 mutations that relieve feedback inhibition or PANK4 deletion. Metabolomic analyses confirm that this rewiring produces an order-of-magnitude increase in intracellular Ac-CoA concentrations (elevating from approximately 0.5 μM to 4–20 μM). Hyperphysiological Ac-CoA levels outcompete small-molecule inhibitors for the catalytic pocket, reducing target occupancy and conferring resistance (136). Beyond target competition, expanded Ac-CoA pools rewire cellular lipid synthesis to alter ferroptosis sensitivity, while extracellular accumulation of metabolic intermediates reshapes the TIME by promoting pro-tumorigenic macrophage phenotypes (e.g., CD206^+^ TAM).

Distinct from catalytic site competition, a second resistance axis operates through the non-catalytic scaffold functions of KAT6A, which are uncoupled from its acetyltransferase activity. KAT6A undergoes LLPS to form biomolecular condensates that physically sequester PARP1. In PARP inhibitor-resistant ovarian cancer models, APEX1-assisted sequestration of PARP1 prevents its recruitment to DNA damage sites, diminishing PARP inhibitor-induced chromatin trapping. Because this sequestration mechanism is independent of KAT6A catalytic activity, catalytic inhibitors (e.g., WM-1119) cannot restore PARP inhibitor sensitivity (19). Crucially, this limitation establishes a compulsory clinical rationale for modalities that achieve structural depletion: complete genetic knockout (KO) and targeted protein degraders (e.g., PROTACs like PRT13722) demonstrate functional equivalence in dismantling non-catalytic scaffolding. Therefore, overcoming LLPS-mediated resistance necessitates transitioning from pure catalytic inhibition toward TPD or allosteric disrupters that dismantle the phase-separation interface.

## 6 Conclusion

Inhibition of KAT6A/B represents a mechanistically grounded but still hypothesis-generating strategy for remodeling the TIME. However, broad clinical translation remains constrained by three major bottlenecks: on-target myelosuppression within normal stem cell compartments, marked genomic context-dependency dictated by TP53 and KAT6B alterations, and acquired resistance driven by Ac-CoA metabolic competition alongside non-catalytic scaffolding functions.

To overcome these translational barriers, future clinical development should adopt a spatiotemporal, hypothesis-driven strategy. Integrating TP53 and KAT6B biomarker selection with next-generation modalities—such as PROTACs or CIPs—may be essential to bypass non-catalytic scaffold-mediated resistance. In parallel, testing hypothesis-generating intermittent dosing schedules may offer a rational path to optimize the therapeutic window between local antitumor immunity and systemic myelosuppression. Delineating the phenotypic divergence between scaffold-preserving catalytic inhibitors and scaffold-dismantling degraders may thus provide a useful framework for clinical candidate optimization.
